# A look-ahead approach to maximizing present value of genetic gains in genomic selection

**DOI:** 10.1093/g3journal/jkac136

**Published:** 2022-06-02

**Authors:** Zerui Zhang, Lizhi Wang

**Affiliations:** Program of Bioinformatics and Computational Biology, Iowa State University, Ames, IA 50011, USA; Department of Statistics, Iowa State University, Ames, IA 50011, USA; Program of Bioinformatics and Computational Biology, Iowa State University, Ames, IA 50011, USA; Department of Industrial and Manufacturing Systems Engineering, Iowa State University, Ames, IA 50011, USA

**Keywords:** genomic selection, present value, time value, mating strategy, heuristic search

## Abstract

Look-ahead selection is a sophisticated yet effective algorithm for genomic selection, which optimizes not only the selection of breeding parents but also mating strategy and resource allocation by anticipating the implications of crosses in a prespecified future target generation. Simulation results using maize datasets have suggested that look-ahead selection is able to significantly accelerate genetic gain in the target generation while maintaining genetic diversity. In this paper, we propose a new algorithm to address the limitations of look-ahead selection, including the difficulty in specifying a meaningful deadline in a continuous breeding process and slow growth of genetic gain in early generations. This new algorithm uses the present value of genetic gains as the breeding objective, converting genetic gains realized in different generations to the current generation using a discount rate, similar to using the interest rate to measure the time value of cash flows incurred at different time points. By using the look-ahead techniques to anticipate the future gametes and thus present value of future genetic gains, this algorithm yields a better trade-off between short-term and long-term benefits. Results from simulation experiments showed that the new algorithm can achieve higher genetic gains in early generations and a continuously growing trajectory as opposed to the look-ahead selection algorithm, which features a slow progress in early generations and a growth spike right before the deadline.

## Introduction

The core of plant breeding is in the selection of breeding parents to improve traits of interest, such as yield, tolerance to environmental stress, resistance to pests, most of which are quantitatively inherited (Wricke and Weber 2010). Traditional selection strategies used to focus on observable phenotype or a handful of assisting markers related to the desired traits. However, these methods are not applicable to polygenic traits, i.e. traits consisting of many small-effect alleles for which effects are scattered and difficult to determine reliably (Jannink *et al.* 2010). With the development of high-throughput genotyping and single nucleotide polymorphism (SNP) effect estimation (Heffner *et al.* 2009; Jannink *et al.* 2010) shedding lights on the field, genomic prediction emerged as a technique for linking genomic information of quantitative traits to phenotypic values. Genomic selection (GS) is an approach to exploiting genomic markers to cater to novel breeding programs and evaluation. In this technique, the genomic estimated breeding value (GEBV), i.e. the sum of the estimated marker effects for a specific individual becomes a popular criterion to evaluate the breeding potential for certain traits without relying on individual’s phenotype (Goddard 2009).

Improvements in genomic prediction models for complex patterns, such as genotype by environment interactions (G × E), have received great attention. Much of the work on GS has been on the design and execution of field trials (Heslot *et al.* 2015; Crossa *et al.* 2017; Hickey *et al.* 2017). What appears to be missing in the body of literature is computational algorithms that use genomic prediction to intelligently select individuals or groups worthy of breeding. These algorithms should be able to not only select optimal breeding parents but also strategically determine the number of crosses to make and progenies to produce under resource and time constraints.

Conventional genomic selection (CGS) selects the individuals with the highest GEBVs as breeding parents (Meuwissen *et al.* 2001), which are assumed to be most likely to produce superior offspring. CGS has been widely adopted in both plant and animal breeding practices due to its simplicity and effectiveness. However, this truncation approach often leads to loss of genetic diversity after only a few breeding cycles. Several more sophisticated selection algorithms have been proposed to address such limitations. Weighted genomic selection modifies the SNP effects with weights to render the inheritance of favorable alleles with low population frequencies (Heffner *et al.* 2010). Optimal haploid value (OHV) evaluates a breeding parent not by its own genetic value but by the genetic value of the best gamete that it can produce in the immediate next generation (Daetwyler *et al.* 2015). OHV also aggregates adjacent SNP markers as recombination blocks distributed across chromosomes to accelerate the computation. Optimal population value (OPV) introduces the concept of group selection and selects a group of breeding parents that possess favorable alleles in complementary loci, thus can produce the best progeny in the long term (Goiffon *et al.* 2017). Look-ahead selection (LAS) extends the concept of OHV and OPV by anticipating the implications of crosses in the current generation on the progeny produced in a prespecific future generation. By selecting optimal crosses to maximize the long-term performance, LAS has been found to be able to not only accelerate genetic gains but also preserve genetic diversity (Moeinizade *et al.* 2019).

Building on the prior work, we propose an algorithm to overcome 2 limitations of LAS, which are the challenges to specify an appropriate deadline in the context of continuous genetic improvement and the weak performance before the deadline. The new algorithm borrows the concept of present value (PV) from the field of finance and uses it to define a new breeding objective, which converts genetic gains in different future generations over a planning window back to the current time using a discount rate, and the trade-off between short-term and long-term performances can be adjusted using the parameters of window length and discount rate. Details of this method are developed in the next section.

## Materials and methods

### Nomenclature

Here, we define some of the parameters and variables used for modeling GS.

**Table:** 

*N*	Number of the individuals in a population, a scalar
*L*	Number of SNPs of an individual, a scalar
*T*	Number of generations in a breeding program, a scalar
*S*	Number of breeding parents to be selected, a scalar
***G***	Genotype of a population, a binary matrix $G\in B^{L\times 2\times N}$, with element $G_{l,m,i}$ indicating whether the allele in the first (*m *=* *1) or second (*m *=* *2) chromosome of diploid individual *i* at locus *l* is a major allele ($G_{l,m,i}=1$) or a minor allele ($G_{l,m,i}=0$)
β	SNP effect, a vector *β* $\in {\mathbb{R}}^{L}$, with *β_l_* being the allele effect for locus *l*
***r***	Recombination frequencies, a vector $r\in {\mathbb{R}}^{L-1}$, with *r_l_* being the recombination frequency between loci *l* and *l *+* *1
***v***	GEBVs, a vector $v\in {\mathbb{R}}^{N}$, with *v_i_* being the GEBV of individual *i*

### Formulation of GS

GS benefits from high-density markers used in whole-genome prediction models, which pave the way for effect estimation of quantitative trait locus for the traits of interest. Here, we use SNPs data, which are a common type of genetic variation among the population. With assumptions of linear additive SNP effects and appropriate high-dimensional point estimation methods, one can model the quantitative relationship between β and the GEBV of individual *i* as $v_{i}=\mu +\sum_{l}\beta_{l}\sum_{m}G_{l,m,i},\forall i\in \{1,\ldots ,N\}$, where *μ* is overall mean (Desta and Ortiz 2014).

With the aforesaid definitions, the goal of GS is to select *S* pairs of breeding parents to achieve specific breeding goals. The general program can be formulated as

(1) $$\underset{x}{\max}\;f(x,G)$$

(2) $$s.t.\;\sum_{i=1}^{N}x_{i}=2S$$

(3) $$\;x_{i}\in \{0,1\}\;i\in \{1,\ldots ,N\}.$$

Here,

- Objective function f(·) represents the genetic breeding value or other appropriately defined breeding objectives. For example, CGS uses the total GEBVs of all selected breeding parents as its objective function: $f(x,G)=\sum_{i}x_{i}v_{i}$.
- Decision variables $x=(x_{i})$ for all i∈{1,…,N} are a binary variable that indicates whether individual *i* is selected $\left(x_{i}=1\right)$ or not $\left(x_{i}=0\right)$, as shown in constraint (3).

### Review of the look-ahead selection algorithm

LAS presents an efficient framework for selecting breeding parents that maximize genetic gain of future progeny in a user-defined deadline generation. It takes into account not only parent selection but also mating, time management, and resource allocation (number of progeny from each cross; Moeinizade *et al.* 2019). The formulation of LAS is shown as follows

(4) $$\underset{x,Y}{\max}\;\varphi$$

(5) s.t. Pr[g(x,Y,G,β,r,T−t) ⩾ φ] ⩾ 1−γ

(6) $$\;\frac{1}{N}\sum_{j=1}^{N}y_{i,j}\;⩽\;x_{i}\;⩽\;\sum_{j=1}^{N}y_{i,j}\;\;i\in \{1,\ldots ,N\}$$

(7) $$\;\sum_{i=1}^{N}\sum_{j=1}^{N}y_{i,j}=2S$$

(8) $$\;y_{i,j}=y_{j,i}\;\;i,j\in \{1,\ldots ,N\}$$

(9) $$\;x_{i},y_{i,j}\in \{0,1\}\;\;i,j\in \{1,\ldots ,N\}.$$

Here,

- decision variable *x_i_* indicates whether individual *i* is selected as a breeding parent (*x_i_* = 1) or not (*x_i_* = 0);
- decision variable $y_{i,j}$ indicates whether individual *i* is mated with *j* ($y_{i,j}=1$) or not ($y_{i,j}=0$);
- parameter t∈{1,…,T} represents the current generation number;
-
φ is the GEBV of the best progeny in the final generation that has a probability of occurrence of at least 1−γ;
- parameter *γ* is a risk tolerance parameter, a larger (smaller) value of which will incentivize the model to maximize the performance in more (less) optimistic scenarios; and
-
g(·) is the GEBV of a random progeny in the final generation *T*, created using the breeding decisions ***x*** and ***Y*** through a look-ahead simulation proposed in Moeinizade *et al.* (2020).

The introduction of the additional decision variable ***Y*** allows the model to further accelerate genetic responses by optimizing mating strategies of the selected individuals (Toro and Varona 2010; Wang *et al.* 2018). Constraint (5) defines φ, which is the *γ* quantile of *g*, the GEBV of a random progeny in the final generation *T*. This constraint also interprets the objective function (4), which is to maximize the genetic gain in the best possible scenario with a probability of at least 1−γ. Constraint (6) ensures that only selected individuals be mated with each other. Constraints (7) and (8) make sure that a total of *S* crosses are made and that the mating is symmetric. Constraint (9) requires all decision variables to be binary.

### Motivation for improvement

Despite the effectiveness of the LAS algorithm in accelerating genetic gain while preserving genetic diversity, it has 2 major limitations that motivated the design of the proposed algorithm.

First, it is challenging to determine an appropriate value for the breeding deadline, since the goal of breeding projects is usually to achieve continuous genetic improvements. To address this limitation, we replace the fixed deadline with a rolling horizon, which restarts its planning timeline over an adjustable time interval. The interval acts as a sliding window to smooth the variability in future offspring and consider the dependence along the breeding process.

Second, the LAS algorithm focuses exclusively on maximizing genetic gain in the terminal generation without considering earlier performance, potentially resulting in unacceptably low short-term genetic gains. In the proposed model, we redefine the breeding objective as the PV of genetic gains over the rolling horizon, which takes a user-defined discount rate, *λ*, accounting for the time value of genetic gains and the market value of successful release of new commercial lines from the breeding program. A genetic gain achieved in generation *τ* would be only $\frac{1}{{\left(1+\lambda \right)}^{\tau }}$ times as valuable as the same genetic gain would have in generation 0. A larger *λ* assigns a higher time value of genetic gain, putting higher weights on shorter-term performances.

**Fig. 1.**
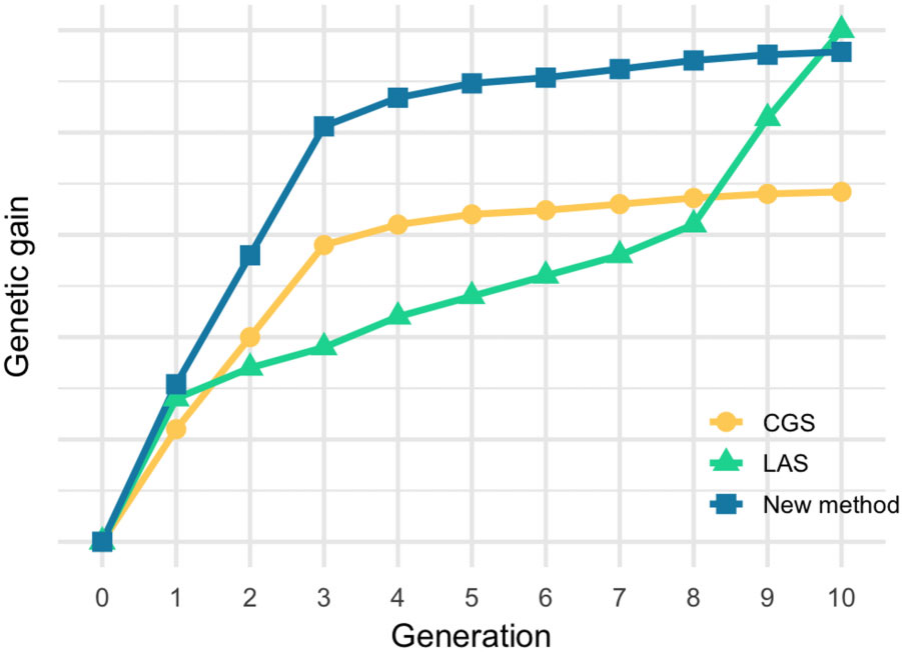
Expected trajectories of genetic gains using the proposed new method, CGS and LAS. The curve for the new method is illustrative, whereas those for the latter 2 methods are from Moeinizade *et al.* (2019). Compared with LAS, the new method is expected to achieve higher genetic gains in earlier generations at the cost of later performance.

As a result, the new method is expected to achieve higher genetic gains in earlier generations albeit at the cost of a weaker performance in the final generation, as illustrated in Fig. 1. This is achieved by maximizing the PV of genetic gains in all generations, with earlier gains having higher values than later ones, similar with the financial concept of “time value of money.” Similar with the compound interest rate, the discount rate can be used to adjust the trade-off between short-term and longer-term genetic gains.

### PV-based look-ahead selection algorithm

We propose to use the PV of GEBVs as the new objective for maximization. In finance (Weitzman 1998; Žižlavskỳ 2014), for a series of cash flows $f_{\tau }$ over certain time period ∀τ∈{1,…,T}, the PV of these cash flows is the summation of their discounted values at time 0: $\sum_{\tau =1}^{T}\frac{f_{\tau }}{{\left(1+\lambda \right)}^{\tau }}$, where *λ* is the compound interest rate, indicating the time value of money. In the context of GS, if we use $\psi_{\tau }$ to denote the GEBV of a progeny in the *τ*th future generation, *W* the number of generations that we look ahead to, and *λ* the discount rate that indicates the “time value of genetic gains,” then the new objective becomes $\sum_{\tau =1}^{W}\frac{\psi_{\tau }}{{\left(1+\lambda \right)}^{\tau }}$. As such, the proposed method, which we refer to as PV-LAS, can be formulated as follows:

(10) $$\underset{x,Y}{\max}\;\zeta (x,Y)=\sum_{\tau =1}^{W}\frac{\psi_{\tau }}{{\left(1+\lambda \right)}^{\tau }}$$

(11) s.t.  Constraints (6), (7), (8), (9)

(12) $$\;Pr\left[g(x,Y,G,\beta ,r,\tau )\;⩾\;\psi_{\tau }\right]\;⩾\;1-\gamma ,\;\tau \in \{1,\ldots ,W\}.$$

Here,

- parameter *W* is the length of the sliding window, indicating the number of generations to look ahead;
- parameter *λ* is the discount rate, indicating the time value of genetic gains. Specifically, the genetic gain $\psi_{\tau }$ in *τ* generations is as valuable as $\frac{\psi_{\tau }}{{\left(1+\lambda \right)}^{\tau }}$ in the current generation; and
-
$\psi_{\tau }$ is the *γ* quantile of a random progeny’ GEBV in the *τ*th future generation given the current decision variables ***x*** and ***Y***.

The model introduces 2 additional parameters: the length of the sliding window *W* and the discount rate *λ*. The window length defines the longest term that the breeders look ahead in the planning horizon, whereas the discount rate determines the trade-off between short-term and long-term genetic gains. A larger (smaller) *λ* places a higher emphasis on shorter-term (longer-term) performances.

PV-LAS can be seen as an extension of LAS with a modified approach to looking ahead. LAS maximizes the performance at a predefined target deadline, which gradually reduces the planning horizon as the breeding process progresses toward the deadline. In contrast, a moving horizon is used in PV-LAS, where the planning horizon is always the length of the sliding window, *W*. As such, PV-LAS is more applicable to breeding programs with goals for continuous genetic improvement. The change in planning horizon requires a different formula to calculate the recombination frequency in a future generation. In particular, equation (13) for LAS in Moeinizade *et al.* (2019) should be modified as the following for PV-LAS.

For all l∈{1,…,L−1}

(13) $${\tilde{R}}_{l}=\{\begin{array}{cc} 0, & \;\text{if}\;\tau \in \{1,2\}\\ \frac{\left(S-1)[1-{\left(1-r_{l}\right)}^{\tau }\right]}{S}, & \;\text{if}\;\tau \in \{3,\ldots ,W\}. \end{array}$$

### Optimization framework

We propose a heuristic search approach to find optimal selection and mating decisions for PV-LAS by iteratively searching the solution space. The workflow of this heuristic algorithm is described as follows:

- **Input**: ***G***, β, ***r***, *W*, *λ*, *S*, *K*, and *γ*.
- **Step 1**: Identify a feasible solution $\left(x^{*},Y^{*}\right)$ and use it as the incumbent solution. Denote the corresponding objective value of the incumbent as $\zeta^{*}$.
- **Step 2**: Randomly choose $\left(\hat{i},\hat{j}\right)$ such that $y_{\hat{i},\hat{j}}z_{\hat{i},\hat{j}}=1$. For all k∈{1,…,N}, evaluate the new solution $\left(\hat{x},\hat{Y}\right)$, which is defined as ${\hat{x}}_{j}=\{\begin{array}{cc} 0 & \text{if}\;j=\hat{j}\\ 1 & \text{if}\;j=k\\ x_{j}^{*} & \text{otherwise} \end{array},\forall j$ and ${\hat{y}}_{i,j}=\{\begin{array}{cc} 0 & \text{if}\;i=\hat{j}\;\text{or}\;j=\hat{j}\\ 1 & \text{if}\;i=\hat{k}\;\text{or}\;j=\hat{k}\\ y_{i,j}^{*} & \text{otherwise} \end{array},\forall (i,j)$. If $\left(\hat{x},\hat{Y}\right)$ is feasible and has a better objective value than the incumbent, then update the incumbent solution $\left(x^{*},Y^{*})\leftarrow (\hat{x},\hat{Y}\right)$ and its corresponding objective value $\zeta^{*}\leftarrow \zeta (\hat{x},\hat{Y})$. Repeat this step until no further improvement of the incumbent can be made.
- **Output**: the locally optimal ***x*** and ***Y***.

From our computational experiences, the LAS algorithm almost always found selfing as the optimal breeding strategy for the last generation. Our explanation for this observation is that when the breeding goal is to maximize the genetic gain in the immediate next generation, the value of a breeding parent can be largely determined by its best gamete that can be produced (within certain risk tolerance). As such, selfing the top #1 breeding parent would be more likely to produce a better progeny than crossing the top #1 with top #2. While this strategy has produced satisfactory results for the case study, it is unnecessarily the optimal strategy for all breeding programs.

### Illustrative example

**Fig. 2.**
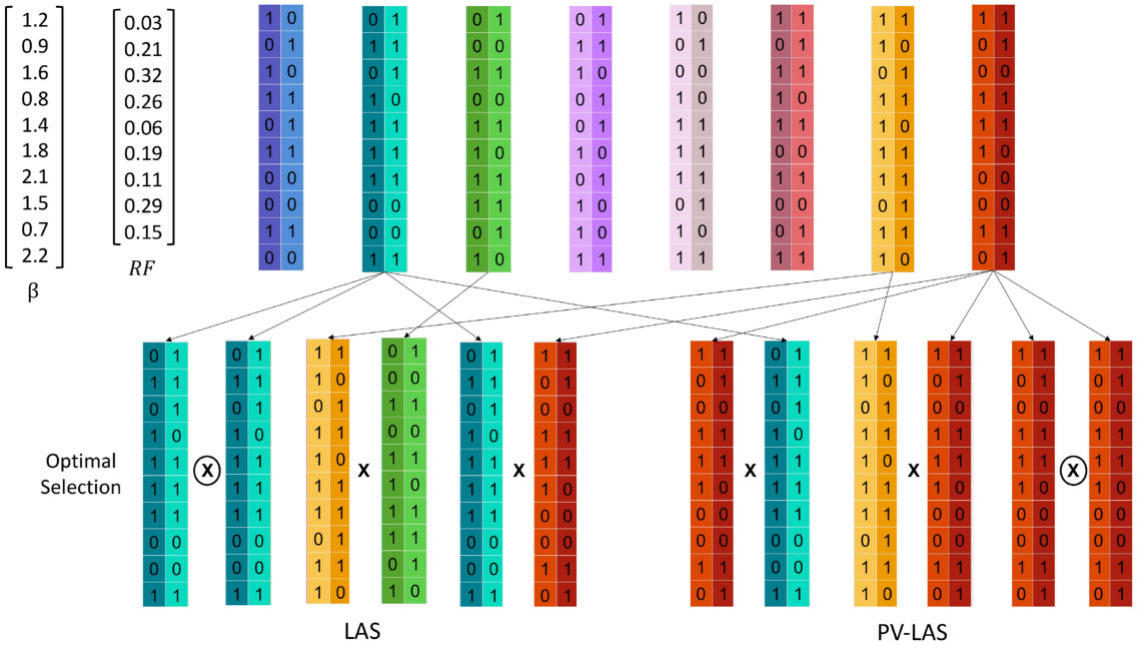
Optimal selection and mating solutions using LAS and PV-LAS for the toy example.

We use a toy example to illustrate the difference between LAS and PV-LAS. Suppose we aim to make *S *=* *3 crosses from a group of *N *=* *8 diploid individuals, each genotyped with *L *=* *10 SNPs. The breeding deadline in LAS and the length of sliding window in PV-LAS were both set as T=W=3. The arbitrarily simulated SNP effect β, recombination frequency and finalized selection and mating results for LAS and PV-LAS are showed in Fig. 2. For each of these 3 future generations, *K *=* *500 future gametes have been simulated. We used γ=0.8.

**Fig. 3.**
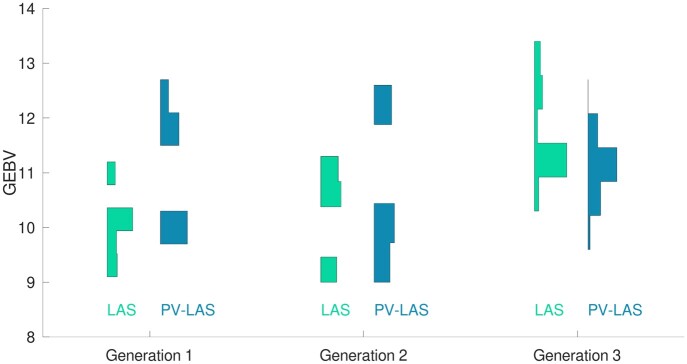
Vertical histograms of GEBVs of 500 random progeny in 3 generations using LAS vs PV-LAS algorithms.

Figure 2 illustrated the solutions given by the 2 methods. Both methods included a cross between the second and the eighth individuals from the left, whereas the remaining 2 crosses were different. Figure 3 shows vertical histograms of GEBVs of 500 random progeny in 3 generations using LAS and PV-LAS, which demonstrated the major differences of these algorithms: LAS resulted in higher genetic gains in the final generation, whereas PV-LAS improved the growth in the first 2 generations with a slightly compromised performance in the third.

## Results and discussion

### Data sources

We conducted computational experiments using the same data set as Moeinizade *et al.* (2019): the genotypic data ***G*** contains genotypes of 369 maize inbred lines consisting of *L *=* *1,406,757 SNPs distributed across 10 maize chromosomes (Goiffon *et al.* 2017). These SNPs were aggregated into 10,000 blocks to increase the computational speed. SNP effects were estimated on the basis of 369 shoot apical meristem phenotypes using the BayesB model (Meuwissen *et al.* 2001; Leiboff *et al.* 2015). Recombination rates were based on the genetic map developed from maize nested association mapping (Yu *et al.* 2008). We assume that the marker effects $\hat{\beta }$ and recombination rates $\hat{r}$ have been estimated reasonably accurately and they stay fixed in our simulations. As a caveat for this simplifying assumption, however, we point out that LAS and PV-LAS are more sensitive to the accuracy of allele effects and recombination frequencies than CGS, because the former 2 extract more information from such data to make more sophisticated decisions.

### Simulation setting

The aim of the simulation was to evaluate the performance of 3 algorithms: CGS, LAS, and PV-LAS with respect to genetic improvement throughout multiple breeding generations. For a group of offspring ***G*** with a size of *N* in at the end of the breeding program, the following criteria were used for evaluating the 3 algorithms:

- Mean of GEBV: $\frac{1}{N}\sum_{i}\sum_{l}\sum_{m}\left(G_{l,m,i}\beta_{l}\right)$. This criterion measures the average performance of genetic gains.
- Lower potential of GEBV: $2\sum_{l}\min_{m,i}(G_{l,m,i}\beta_{l})$. This criterion gives the theoretical lower bound of GEBV based on the remaining genetic diversity.
- Upper potential of GEBV: $2\sum_{l}{\text{max}}_{m,i}(G_{l,m,i}\beta_{l})$. This criterion gives the theoretical upper bound of GEBV based on the remaining genetic diversity.
- PV of GEBV: $\sum_{\tau =1}^{T}\underset{i}{\max}\sum_{l}\sum_{m}\frac{G_{l,m,i}^{\tau }\beta_{l}}{{\left(1+\lambda \right)}^{\tau }}$. This criterion measures the PV of GEBVs over a period of *T* generations for a given discount rate *λ*.

Each of the simulations consisted of 3 steps: initialization, selection, and reproduction. In the initialization step, *N *=* *200 individuals were randomly selected among the given 369 maize inbred lines. In the selection step, GS algorithms were used to select the optimal crosses. In the reproduction step, crosses from the previous step are made, each producing *N *=* *200 progeny, and then it goes back to the selection step for the next generation until the final generation *T *=* *10. Each algorithm was tested for 500 independent simulations. Parameters λ=0.1 and γ=0.8 were used for all experiments.

- For CGS, *S* = 10 pairs of individuals with the highest GEBVs were selected and randomly mated to make 10 crosses, each producing 20 progenies.
- For LAS, *S* = 10 pairs of individuals were selected and mated.
- For PV-LAS, *S* = 10 pairs of individuals were selected and mated.

### Performance comparison

**Fig. 4.**
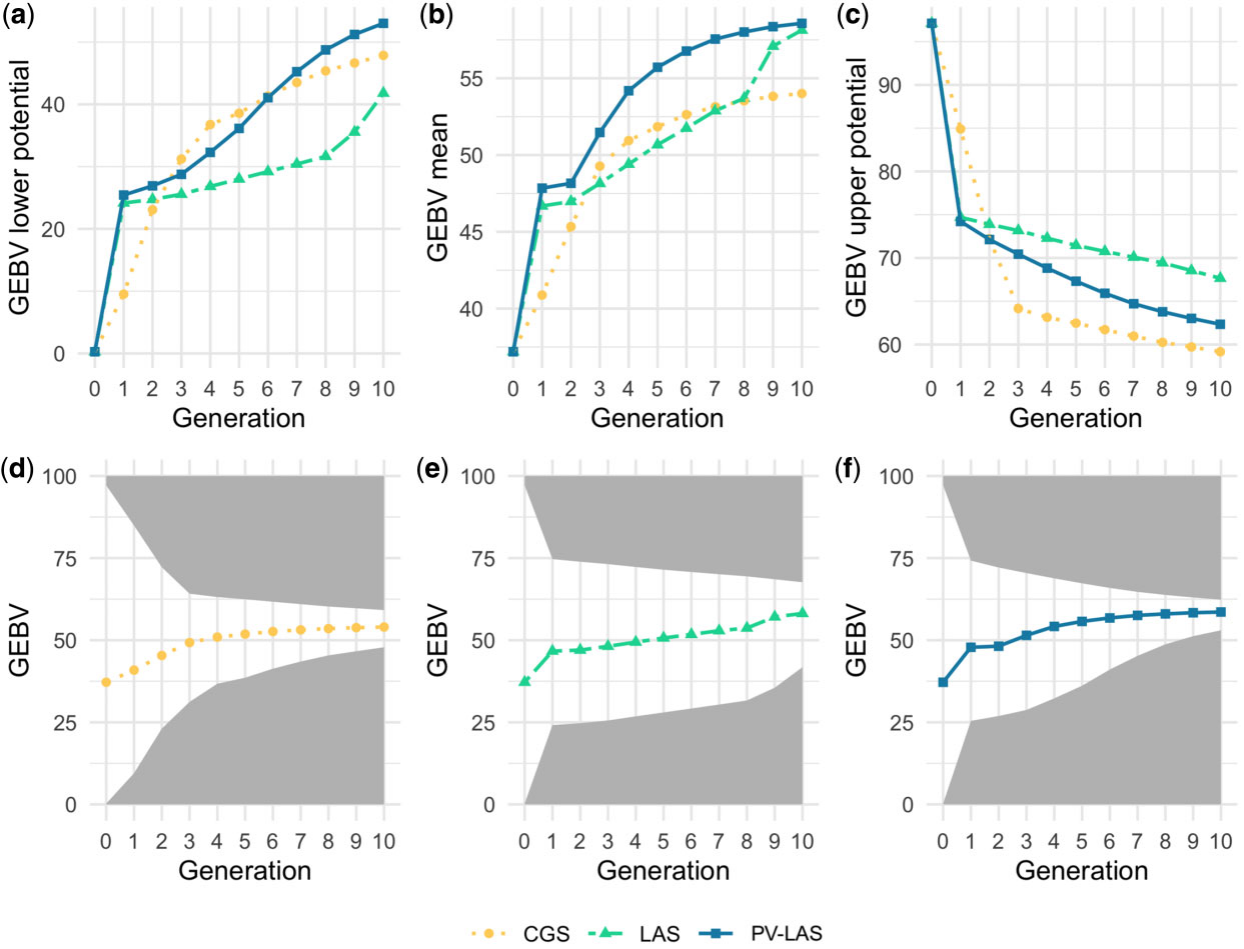
Comparison of CGS, LAS, and PV-LAS over 10 generations with respect to 3 criteria calculated based on the average of 500 simulations: (a) GEBV lower potentials, (b) GEBV mean, and (c) GEBV higher potential. All 3 criteria were also plotted together in subfigures (d), (e), and (f) for the 3 selection methods.

Results for genetic gain comparison are shown in Fig. 4. The GEBV mean in Fig. 4b matched our expected trajectory in Fig. 1. Figure 4, a and c showed how PV-LAS compromised genetic diversity, with respect to LAS, to achieve higher growth in early generations, although PV-LAS still largely outperformed CGS in both genetic gain and genetic diversity. Figure 4, d–f showed mean, lower potential, and higher potential of GEBV together for the 3 selection methods. Although LAS and PV-LAS use a more forward-looking selection strategy than CGS, they have narrower ranges between upper and lower bounds in the first 2 generations. This counter-intuitive result is because they only select parents whose favorable alleles can be aggregated within time and resource constraints, which means that some otherwise high-performing parents may be discarded if their favorable alleles require more time or resources than available to be integrated with the selected ones. On the other hand, CGS will select all high-performing parents without anticipation of their future progeny, which may lead to higher genetic diversity in the first generations but inevitable loss of genetic gain and diversity in the longer term.

**Fig. 5.**
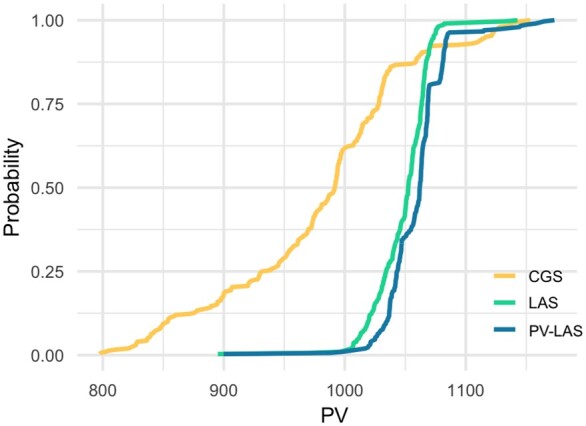
Cumulative distribution functions of PVs for CGS, LAS, and PV-LAS.

Figure 5 compares the empirical cumulative distribution functions of the PVs of GEBVs resulted from different selection methods. We observe that PV-LAS is on the right-hand side of LAS in almost all quantiles, which indicates the stochastic dominance of PV-LAS over LAS in terms of the PV of genetic gains. This was expected because PV-LAS was designed to optimize the PV of GEBVs.

### Sensitivity analysis on PV-LAS parameters

**Fig. 6.**
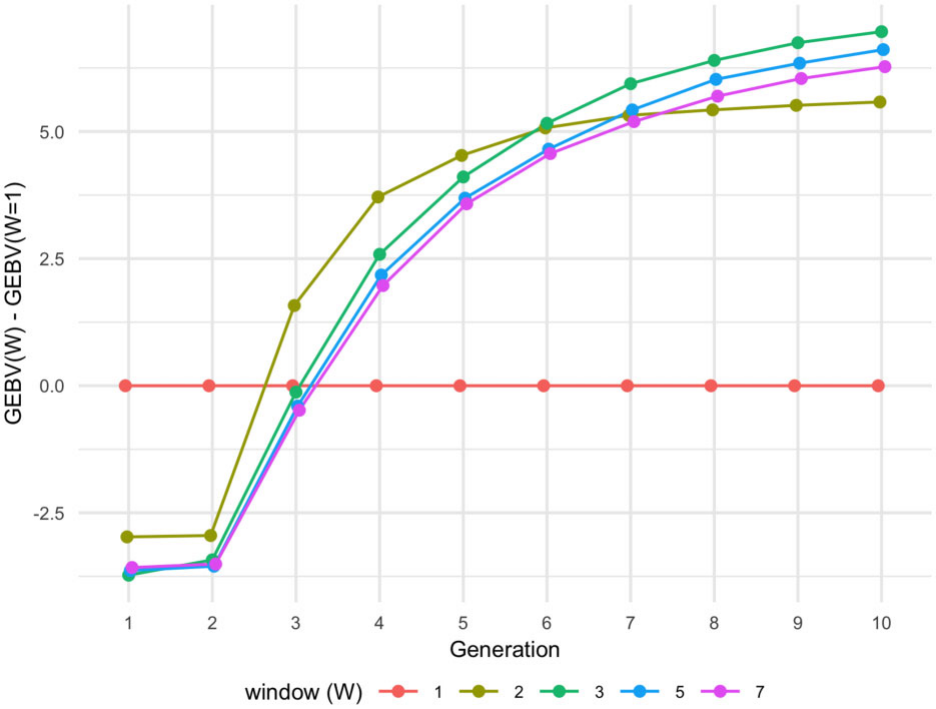
Differences in genetic gains between the benchmark case of *W *=* *1 and other window lengths for PV-LAS, averaged over 500 independent experiments.

Sensitivity analysis was performed to test the influence of window length *W* and discount rate *λ* on the performance of PV-LAS. Figure 6 shows the average (over 500 independent experiments) differences in genetic gains between the benchmark case of *W *=* *1 and other window lengths. When *W *=* *1, the model focused on genetic gain in the immediate next generation; as a result, GEBVs jumped to a plateau after the first selection and lost diversity and potential for future growth. The figure shows that the effect of *W* is not monotonic and that a balance between short-term and long-term growth requires a window length that is neither too short nor too long. For this particular case study, *W *=* *3 achieved the best performance between generations 6 and 10, but it is unnecessarily optimal for other studies or datasets. In general, the sensitivity analysis should be done for each new dataset to identify the best set of parameters.

**Fig. 7.**
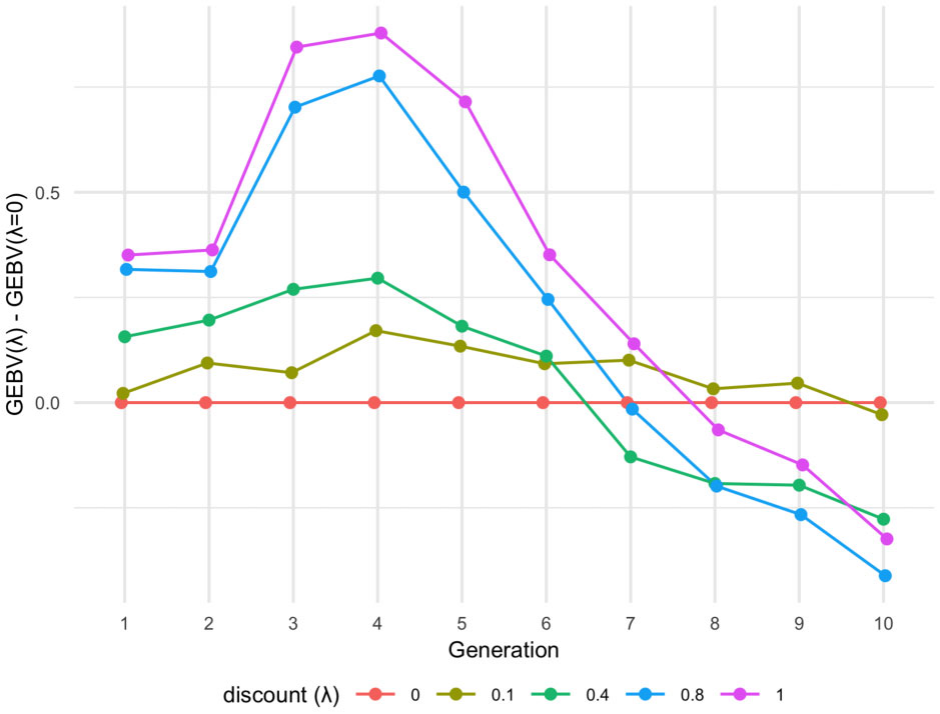
Differences in genetic gains between the benchmark case of *λ* = 0 and other discount rates for PV-LAS, averaged over 500 independent experiments. Window length is fixed at *W *=* *3.

Figure 7 shows the differences in genetic gains between the benchmark case of *λ* = 0 and other discount rates, with window length fixed at *W *=* *3. When *λ* = 0, the model focused on the nominal genetic gain and ignored its time value. Larger (smaller) *λ* values put higher (lower) emphasis on the time value of genetic gain and led to higher (lower) growth in the short-term and weaker (stronger) performance in later generations.

## Conclusion

The introduction of the PV concept to GS is central to this work. PV-LAS uses the PV of GEBVs over a certain window period as the breeding objective by discounting genetic gain in future generations back to the current time. As such it balances the short-term and long-term benefits of genetic growth and provides a continuous growth trajectory. At the same time, PV-LAS makes a moderate compromise on genetic diversity in order to achieve a higher growth in early generations.

Computational results demonstrated the effectiveness of the new algorithm. Optimal window length *W* and discount rate *λ* for specific data sets can be determined using sensitive analyses.

Several research directions are worthy of future investigation. First, the simulations in this paper were based on the assumption that the estimates of additive effects of SNPs and recombination frequencies are reasonably accurate. Analysis should be conducted to assess the effects of inaccurate estimations and how to mitigate such effects. Second, we can extend PV-LAS to accommodate both additive and nonadditive effects, such as dominance and epistasis effects. Third, PV-LAS does not explicitly address G × E, which can often be substantial and create problems in finding consistently superior genotypes, leading to reduced heritability and overall genetic gain. Future research should focus on the convergence of crop modeling and machine learning approaches to explore more advanced strategies to address G × E in the breeding process.

## Data availability

The datasets used the computational experiments were derived from sources in the public domain as described in *Data Sources*.
